# Platelet‐borne complement proteins and their role in platelet–bacteria interactions

**DOI:** 10.1111/jth.13495

**Published:** 2016-11-11

**Authors:** I. Arbesu, M. Bucsaiova, M. B. Fischer, C. Mannhalter

**Affiliations:** ^1^ Department of Laboratory MedicineMedical UniversityViennaAustria; ^2^ Center for Biomedical TechnologyDonau‐Universität KremsKremsAustria; ^3^ Department of Blood Serology and Transfusion MedicineMedical University of ViennaViennaAustria

**Keywords:** complement, Gram‐negative bacteria, infection, megakaryocytes, platelets

## Abstract

**Essentials**

- Platelets play an important role in pathogen recognition.
- Platelets contain several complement factors and can interact with *E. coli*.
- Platelet's complement protein C3 differs from plasmatic C3 in its electrophoretic mobility.
- Upon contact with bacteria, platelets are activated and can enhance complement activation.

**Summary:**

**Background**

The role of platelets in immune defense is increasingly being recognized. Platelets bind complement proteins from plasma, initiate complement activation, and interact with bacteria. However, the contribution of platelets to complement‐mediated defense against bacterial infections is not known in detail.

**Objectives**

To assess platelet interactions with *Escherichia coli* strains, and evaluate the contributions of platelet complement proteins to host defense.

**Methods**

We studied the cell–cell interactions of a pathogenic and a non‐pathogenic *E. coli* strain with platelet concentrates, washed platelets and manually isolated platelets by flow cytometry and ELISA. The presence of complement proteins and complement RNA in megakaryocytes and platelets was analyzed by PCR, RT‐PCR, confocal microscopy, and western blotting.

**Results**

Incubation with *E. coli* leads to platelet activation, as indicated by the expression of CD62P and CD63 on the platelet surface. RNA and protein analyses show that megakaryocytes and platelets contain complement C3, and that platelet C3 migrates differently on polyacrylamide gels than plasmatic C3. Activation of platelets by bacteria leads to translocation of C3 to the cell surface. This translocation is not induced by thrombin receptor activating peptide or lipopolysaccharide. Interaction of platelets with *E. coli* occurs even in the absence of plasma proteins, and is independent of platelet toll‐like receptor 4 and α_2b_β_3_ (glycoprotein IIbIIIa).

**Conclusion**

Platelets contain a specific form of C3. Importantly, they can modulate immune defense against bacteria by enhancing plasmatic complement activation.

## Introduction

Platelets are blood cell elements without a nucleus that are derived from bone marrow megakaryocytes. They constitute the second most abundant cell type in the circulation. During thrombopoiesis, they receive proteins, organelles, granules and RNA from megakaryocytes. Besides their role in hemostasis, platelets are important in inflammation, and are connected to cellular and humoral components of the immune system. Three mechanisms of interaction between platelets and bacteria have been described: (i) bacteria bind directly to platelet receptors such as toll‐like receptor (TLR) 2, TLR4, and glycoprotein (GP) Ibα 1, 2; (ii) bacteria bind plasma proteins, e.g. fibrinogen, von Willebrand factor, and IgG, which then interact with platelet receptors (GPIIbIIIa, GPIbα, and FcγRIIa) 3, 4; and (iii) platelets respond to toxins released by bacteria, such as α‐toxin, Shiga toxin and lipoteichoic acid by undergoing apoptosis or forming platelet aggregates 5, 6, 7. Following direct or indirect interaction, bacteria can cause platelet activation, platelet aggregation, granule release, and the formation of platelet–leukocyte complexes. Analyses of Gram‐positive bacteria such as *Staphycococcus aureus* and *Streptococcus sanguis* showed all types of interaction, involving multiple bacterial proteins and platelet receptors 8, 9, 10, 11, 12. Gram‐negative bacteria are less well studied, and are thought to interact with platelets via platelet TLR4 3. Different types of *Escherichia coli* lipopolysaccharide (LPS) stimulate the production of cytokines in platelets, cause neutrophil recruitment to sites of infection, and promote the formation of neutrophil extracellular traps, resulting in bacterial clearance 13, 14, 15. However, *in vitro* data regarding the effect of *E. coli* LPS on platelet activation and aggregation are controversial 13, 16, 17, 18.

Platelets have been shown to interact with the complement system, which comprises several plasmatic proteins with immunologic and inflammatory properties. Among their various surface proteins, platelets contain several complement receptors, such as cC1qR 19, gC1qR 20, 21, C3aR 22, 23, and C5aR 24, as well as P‐selectin 2, 25. Platelets bind plasma complement proteins via complement receptors, whereby they become activated 25. Activated platelets (e.g. after thrombin activation) can activate the complement cascade 26. Platelets also express complement regulatory molecules such as CD59, factor H, and decay acceleration factor, which prevent excessive complement activation on the platelet surface 27, 28, 29. The importance of platelet–complement interactions has been studied in hemolytic uremic syndrome caused by Shiga toxin‐producing *E. coli* infection 30. After exposure to Shiga toxin, platelet microparticles and platelet–leukocyte complexes carry high levels of surface‐bound C3 and C9, which may contribute to a prothrombotic state and organ damage. Studies with *Listeria monocytogenes* showed that bacterial clearance was dependent on platelets, and involved plasmatic C3 and platelet GPIb 31. High‐throughput analyses showed that platelets contain complement RNA and proteins 32, 33. Possibly, these intracellular complement factors support platelet function as pathogen ‘sensors’ in the fight against dangerous intruders.

We evaluated whether complement proteins (C3 and C5) are synthesized in megakaryocytes and are stored in platelets intracellularly. We investigated whether this complement C3 is retained in platelets, or is activated and released upon contact of platelets with bacteria. We also studied whether and under which conditions platelet complement products support defense against bacteria, and if and how platelets influence complement activation in plasma in the presence of *E. coli*.

## Materials and methods

The study was approved by the Ethics Committee of the Medical University of Vienna.

### Megakaryocytes and cell lines

Cord blood was obtained from the Clinic of Gynecology of the Medical University of Vienna. All donors gave their written informed consent. CD34^+^ hematopoietic stem cells were isolated from cord blood with CD34 MACS magnetic beads (Miltenyi, Bergisch Gladbach, Germany). CD34^+^ cells were cultured with 50 ng mL^−1^ thrombopoietin, 1 ng mL^−1^ stem cell factor and interleukin‐3 (Miltenyi) in Stem Pro 34 medium (Invitrogen, Carlsbad, CA, USA) at 37 °C in 5% CO_2_ for 12 days to obtain mature culture differentiated megakaryocytes. Mature megakaryocytes were stained with CD41, CD14 and CD45 antibodies (eBioscience, San Diego, CA, USA), and their purity was analyzed by fluorescence‐activated cell sorting (FACS). Cultures containing > 3% contaminating leukocytes were used for our experiments. Cell lines CHRF, Meg‐01, HL‐60 and HepG2 were purchased from ATCC. They were grown in RPMI medium or Dulbecco's modified Eagles's medium (Invitrogen) supplemented with 10% fetal bovine serum and gentamicin (Gibco, Carlsbad, CA, USA) at 37 °C in 5% CO_2_.

### Platelets

Platelet concentrates (PCs) from healthy donors were obtained by apheresis with the Trima Accel automated blood collection system. PCs were leukocyte‐depleted by use of the LRS chamber to a concentration of < 1 leukocyte per 10^5^ platelets. PCs containing ~ 5% plasma were used directly (platelet concentrates) or were washed twice with SSP+ (Macopharma SA, Turcoing, France) containing 400 nm prostaglandin I_2_ (PGI_2_) (Sigma‐Aldrich, Munich, Germany) to remove residual plasma (washed platelet concentrates).

Manually isolated platelets from healthy donors were prepared from fresh citrated blood collected at the Department of Transfusion Medicine, Medical University of Vienna. All donors gave their written informed consent. The blood was centrifuged within 30 min at room temperature at 150 × *g* for 15 min to obtain platelet‐rich plasma (PRP). This was mixed with Optiprep (Axis‐Shield, Oslo, Norway), and subjected to centrifugation at 300 × *g* for 15 min. The platelet layer was recovered, resuspended in HEPES–Tyrode buffer (10 mm HEPES, 137 mm NaCl, 2.8 mm KCl, 1 mm MgCl_2_, 12 mm NaHCO_3_, 0.4 mm Na_2_HPO_4_, 5.5 mm glucose, and 0.35% bovine serum albumin [BSA]), and centrifuged at 800 × *g* for 10 min. The platelet pellet was washed with HEPES–Tyrode buffer, centrifuged at 500 × *g* for 10 min, and resuspended in HEPES–Tyrode buffer or SSP+ (69.3 mm NaCl, 10.8 mm trisodium citrate, 32.5 mm sodium acetate, 28.2 mm phosphate, 5 mm KCl, and 1.5 mm magnesium). The platelet count was determined on a Sysmex XE‐2100 instrument (Sysmex, Kobe, Japan). Platelets were allowed to rest for 1 h before experiments were performed. All centrifugations were carried out without brake in the presence of 400 nm PGI_2_ (Sigma‐Aldrich). In all isolations, the contaminating number of leukocytes was < 1 leukocyte per 4 × 10^4^ platelets.

For some experiments, citrated plasma was prepared by centrifugation of blood at 2000 × *g* for 20 min at 4 °C without brake followed by a second centrifugation for 10 min at 18 000 × *g* at 4 °C to obtain platelet‐free plasma, which was aliquoted and immediately frozen at − 80 °C.

### Bacteria

The uropathogenic complement‐resistant *E. coli* O18:K1 bacterial strain was isolated from a patient and was a gift from S. Knapp (Medical University Vienna, Austria). The non‐pathogenic *E. coli* K12 C600 strain was purchased from the Coli Genetic Stock Center (Yale, CT, USA). Bacteria were grown in lysogeny broth medium at 37 °C until they reached an OD_600 nm_ of 0.4–0.9. They were either collected by centrifugation at 18 000 × *g* for 10 min, resuspended in SSP+ buffer, and used immediately for the experiments, or killed by heating at 80 °C for 90 min (heat‐killed [HK]). Bacteria were washed with 0.95% NaCl, adjusted to 4.7 × 10^8^ cells mL^−1^, aliquoted in SSP+ buffer, and stored at − 20 °C for later use. Bacterial viability after heat killing was evaluated by plating the bacteria at 37 °C in agar plates overnight.

### Analysis of platelet–bacteria interactions by flow cytometry

Platelets from PRP or manually isolated platelets (1 × 10^6^) were resuspended in SSP+ in the presence of 1.25 mm Gly‐Pro‐Arg‐Pro peptide (Sigma‐Aldrich). Platelets were exposed to *E. coli* at a 1 : 10 ratio (live or heat‐inactivated). To analyze LPS from *E. coli* O111:B4 (Sigma‐Aldrich), 3 μg mL^−1^ LPS was incubated with 1 × 10^6^ platelets at 37 °C for 45 min and 3 h. In control experiments, TLR4 was blocked by incubation with 20 μg mL^−1^ polyclonal antibody nTLR4 (Invitrogen), and GPIIbIIIa receptors were blocked with 1–2 mm Arg‐Gly‐Asp‐Ser (RGDS) peptide 34 (Tocris Bioscience, Bristol, UK), for 20 min before incubation with bacteria. To block the FcγRII receptor on platelets, AT10 at 4 μg mL^−1^ (AbD Serotec, Oxford, UK) was used, which prevented binding of IgG antibodies. Control experiments were performed with platelets activated with 14 μm thrombin receptor activating peptide 6 (TRAP‐6), 5 μm ADP (Roche, Penzberg, Germany), or 1 U mL^−1^ thrombin (Sigma‐Aldrich).

Stimulation of platelets was stopped by addition of 1% formalin (Scharlab, Barcelona, Spain). Cells were washed with phosphate‐buffered saline (PBS), and exposed to monoclonal CD41–allophycocyanin (APC), CD63–fluorescein isothiocyanate (FITC), CD62P–phycoerythrin (BioLegend, San Diego, CA, USA) and CD61–FITC (eBioscience), or polyclonal C3d and C3c–FITC (Dako, Glostrup, Denmark). Flow cytometry was performed within 3 days on a FACScalibur (BD Biosciences, San Jose, CA, USA), and analyses were performed with flowjo (TreeStar, Ashland, OR, USA).

### Protein analyses of megakaryocytes and platelets following exposure to bacteria

Mature megakaryocytes differentiated in culture (1 × 10^6^) were exposed to live and HK *E. coli* O18:K1 and *E. coli* K12 C600 at a concentration of 4.7 × 10^7^ colony‐forming units mL^−1^, in the absence of plasma or other sources of complement proteins, for 15 min, 1 h, 3 h, or overnight. Manually isolated platelets, washed platelets from concentrates or PCs prepared for therapeutic use containing ~ 5% plasma were incubated with bacteria under the same conditions, at a platelet/bacteria ratio of 2 : 1. After incubation, the platelet–bacteria suspension was centrifuged at 2000 × *g* for 15 min. Supernatants were removed, aliquoted, and frozen at − 80 °C. The cell pellets were washed twice with PBS (Sigma‐Aldrich), and lysed with radio‐immunoprecipitation (RIPA) buffer containing SDS and a protease inhibitor cocktail (Thermo Scientific, Boston, MA, USA). Supernatants and lysates were used for ELISA analyses. In certain experiments, bacterial cells and platelets were separated by centrifugation at 18 000 × *g* for 10 min.

For some ELISA experiments, bacteria were incubated at room temperature for 1 h with plasma. Supernatants were then collected and incubated with (i) manually isolated platelets, (ii) manually isolated platelets that had been previously activated by 14 μm TRAP‐6, or (iii) *E. coli* K12 C600 for 1 h, or (iv) platelets treated with 300 μm theophylline and 500 μm adenosine to block platelet activation.

Quantitative analyses of the activation of complement proteins in supernatants and lysates were performed with C3a and C5a ELISA kits (Quidel, San Diego, CA, USA).

### Western blot protein analyses

Platelets, megakaryocytes, HepG2 cells and HL‐60 cells were lysed in RIPA buffer. After determination of the protein concentration by bicinchoninic acid analysis (Thermo Scientific), 18 μg of total protein was mixed with 100 mm dithiothreitol and Nupage loading buffer (Invitrogen), and boiled for 5 min. The presence of complement proteins was tested for with reducing SDS‐PAGE on 6–7% polyacrylamide gels under standard conditions. Proteins were transferred to nitrocellulose membranes, which were blocked with 5% skimmed milk in PBS containing 0.1% Tween‐20 prior to an overnight incubation with C3c–horseradish peroxidase (HRP) sheep polyclonal antibody (1 : 1000; Life Span Biosciences, Seattle, WA, USA) or C3d rabbit polyclonal antibody (1 : 3000; Dako). Goat anti‐rabbit H + L HRP (1 : 50 000; BioRad, Hercules, CA, USA) was used as the secondary antibody, and the signal was developed with the ECL West Pico and West Femto detection system (Thermo Scientific).

### Immunocytochemistry

For immunocytochemistry, cytospins of megakaryocytes were prepared according to standard procedures. They were stained with C3c chicken polyclonal antibody (1 : 300; Antibodies Online, Aachen, Germany), C3d rabbit polyclonal antibody (1 : 150, Dako), anti‐rabbit APC (Invitrogen, Life Technologies, Carlsbad, CA, USA), and CD61–FITC (1 : 200; eBioscience), or isotypes in 3% PBS 0.1% BSA and Triton X‐100 for 1 h at room temperature. After being washed in PBS, the slides were stained with secondary donkey anti‐chicken Cy5 antibody (1 : 500; Jackson Immuno Research, West Grove, PA, USA) or goat anti‐rabbit AF555 (1 : 2000; Invitrogen) for 45 min at room temperature. Platelets from PCs or resting platelets were attached to polylysine‐coated glass slides in the presence of 1 μm PGI_2_ (Sigma‐Aldrich), before being stained with C3c, C3d and CD61 antibodies. An LSM 700 confocal microscope (Carl Zeiss, Jena, Germany) with a × 63 objective and zen 2009 black software were used for the immunocytochemical analysis.

### RNA isolation and analysis

RNA from megakaryocytes was obtained with an RNAeasy kit (Qiagen, Hilden, Germany), according to the manufacturer's instructions. Platelet RNA was isolated with a standard phenol–chloroform procedure. For RNA isolation from PCs, 1 × 10^9^ platelets were lysed with 1 mL of Trizol (Invitrogen). Cell lysates were transferred to a Phase Lock Tube (Prime‐5) together with chloroform, and were centrifuged at 4 °C for 10 min at 13 000 × *g*. Isolated RNA was treated with DNase I according to the manufacturer's instructions (Invitrogen). cDNA was synthesized with the High Capacity cDNA reverse transcription kit with RNase inhibitor (Invitrogen).

The presence of C3 and C5 mRNA in platelets at different time points was determined by quantitative real‐time PCR with TaqMan probes (Applied BioSystems, Foster City, CA, USA). An initial denaturation cycle of 10 min at 95 °C was followed by 50 cycles of 15 s at 95 °C and 1 min at 60 °C. In megakaryocytes, mRNA expression was analyzed by qualitative PCR performed in an Eppendorf Vapo Protect thermo cycler. For this, an initial denaturation cycle of 10 min at 95 °C was followed by 35 cycles of 1 min at 95 °C, 1 min at 63 °C, and 1 min at 75 °C; a final elongation of 10 min at 72 °C concluded the reaction. The following intron‐spanning primers were used: exon 9 C3 forward, 5′‐TGACATGGTGCAGGCAGAGCG‐3′; exon 10 reverse, 5′‐CCACGGGGACTCGGTAGGCT‐3′; exon 13 C5 forward, 5′‐TCCACTTTGGCACGAGGGAGA‐3′; and exon 14 reverse, 5′‐CCACTGCTGCTAATGCCACCCA‐3′.

### Data analysis

The results of at least three experiments are presented as mean ± standard error of the mean. Differences were assessed with unpaired *t*‐tests between groups and in comparison with controls. *P*‐values of < 0.05 were considered to be statistically significant. Three levels of significance were differentiated: *P* < 0.05, *P* < 0.01, and *P* < 0.001.

## Results

### Platelets are activated by *E. coli*

**Figure 1:**
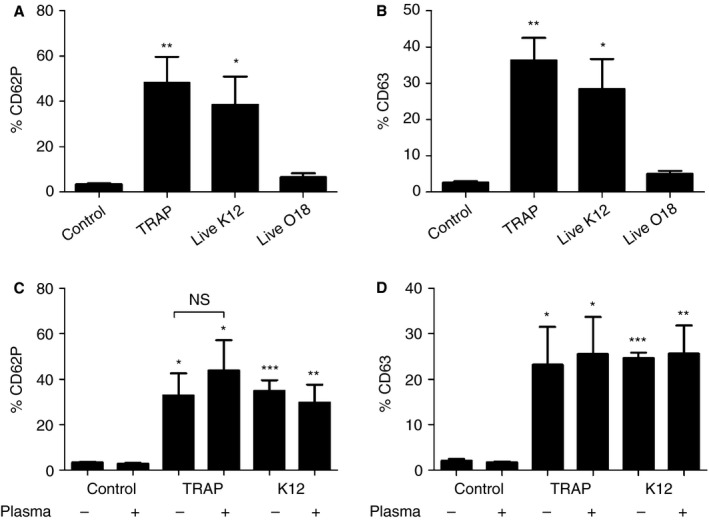
CD62P and CD63 expression on platelets after incubation with bacteria. (A, B) Detection of CD62P (A) and CD63 (B) in platelet‐rich plasma platelets by flow cytometry, following gating for CD41^+^ platelets that were either unstimulated, stimulated with thrombin receptor activating peptide 6 (TRAP‐6) or stimulated with live *Escherichia coli* K12 or live *E. coli* O18:K1 for 45 min (*n* = 4). (C, D) CD62P expression (C) and CD63 expression (D) were also present in manually isolated platelets that were either unstimulated, stimulated with TRAP‐6 or stimulated with live *E. coli* K12 or live *E. coli* O18:K1 for 45 min in the presence or absence of 5% autologous plasma (*n* = 5). Data represent percentages (mean ± standard error of the mean) from independent experiments. Levels of significance with respect to the controls are as follows: *P < 0.05, *P < 0.01, and ***P < 0.001. NS, not significant.

**Figure 2:**
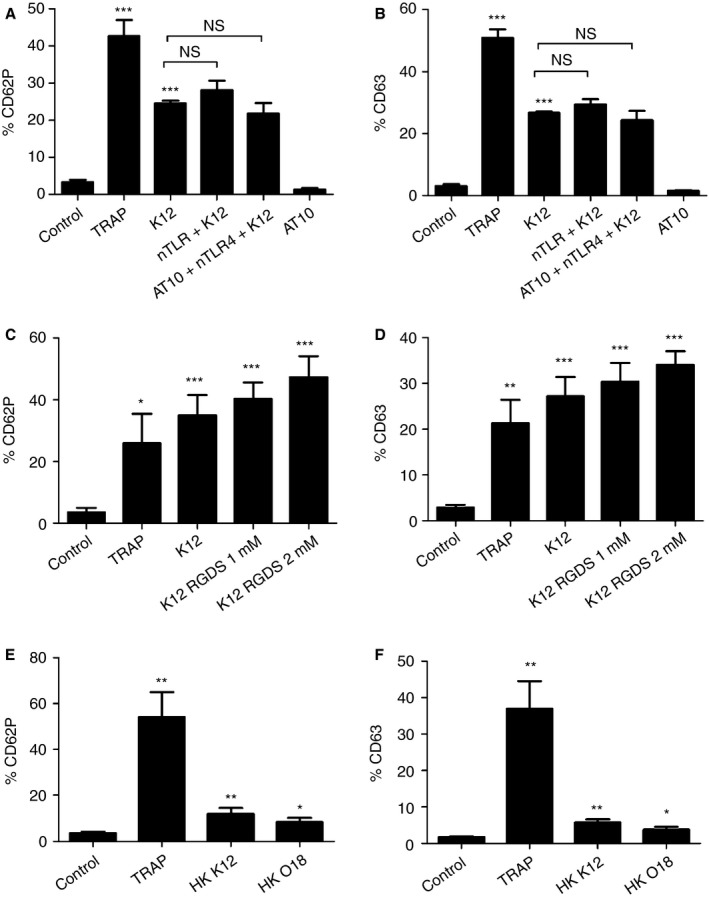
*Escherichia coli* activates platelets independently of toll‐like receptor (TLR) 4 and glycoprotein (GP) IIbIIIa. (A, B) Before stimulation with various agonists, platelets were incubated with 20 μg mL^−1^ neutralizing polyclonal TLR4 antibody for 20 min. AT10 was used to block FcγRII. Stimulation was performed with thrombin receptor activating peptide 6 (TRAP‐6) or live *E. coli* K12. CD62P (A) and CD63 (B) expression was analyzed by flow cytometry (*n* = 3). Unstimulated platelets were used as controls. Bars represent percentages (mean ± standard error of the mean [SEM]). (C, D) Prior to stimulation with various agonists, platelets were also incubated with 1–2 mm Arg‐Gly‐Asp‐Ser (RGDS) peptide to block the GPIIbIIIa receptor. Stimulation was performed with TRAP‐6 or live *E. coli* K12. CD62P (C) and CD63 (D) expression was subsequently analyzed (*n* = 6). Unstimulated platelets were used as controls. Bars represent percentages (mean ± SEM). (E) Platelet‐rich plasma (2.5 μL) was incubated with heat‐killed (HK) *E. coli* K12 and HK *E. coli* O18:K1, and compared with unstimulated and TRAP‐stimulated platelets. Expression of CD62P (E) and CD63 (F) is shown. The data represent percentages (mean ± SEM) from six independent experiments. Levels of significance with respect to the controls are as follows: *P < 0.05, *P < 0.01, and ***P < 0.001. NS, not significant.

We found that incubation with *E. coli* K12 C600 led to platelet activation in PRP, as demonstrated by the expression of CD63 and CD62P on the platelet surface. Manually isolated washed platelets were also activated by *E. coli* K12 C600. Platelet activation by *E. coli* K12 C600 was comparable to the activation achieved with TRAP‐6 (Fig. 1A–D). The complement‐resistant *E. coli* O18:K1 strain was not able to induce significant platelet activation in PRP (Fig. 1A,B). Mean fluorescence intensity (MFI) results are shown in Fig. S1A–D. To determine whether the activation of platelets by *E. coli* K12 C600 was mediated by platelet TLR4 15, PRP and washed platelets were incubated with 1 μg mL^−1^ and 3 μg mL^−1^ *E. coli* O111:B4 LPS. In agreement with the literature 13, 35, 36, platelets were not activated by LPS (data not shown). Pretreatment of platelets with a polyclonal anti‐TLR4 antibody prior to exposure to *E. coli* K12 C600 did not inhibit platelet activation. Possible platelet activation via binding of nTLR4 to the Fc receptor was blocked with AT10. These data suggest that this *E. coli* strain does not interact with platelets via the TLR4 receptor (Fig. 2A,B). Integrin α_2b_β_3_ is the most abundant GP on platelets. It has been reported to mediate interactions of platelets with bacteria 37, 38. Therefore, the integrin‐binding capacity was inhibited with 1 mm and 2 mm of an RGDS peptide. Incubation of the pretreated platelets with bacteria did not reduce platelet activation (Fig. 2C,D). MFI data are shown in Fig. S2A–F.

FACS analyses showed that HK *E. coli* K12 C600 was able to trigger platelet activation to a level of approximately one‐third of that induced by live *E. coli* (Table S6). Interestingly, HK *E. col*i O18:K1 also activated platelets, as indicated by increased CD62P levels (Fig. 2E,F). Representative scatter plots of FACS analyses are shown in Figs S3–S7. Statistical data are shown in Tables S1–S8.

### Megakaryocytes and platelets contain complement RNA and protein

**Figure 3:**
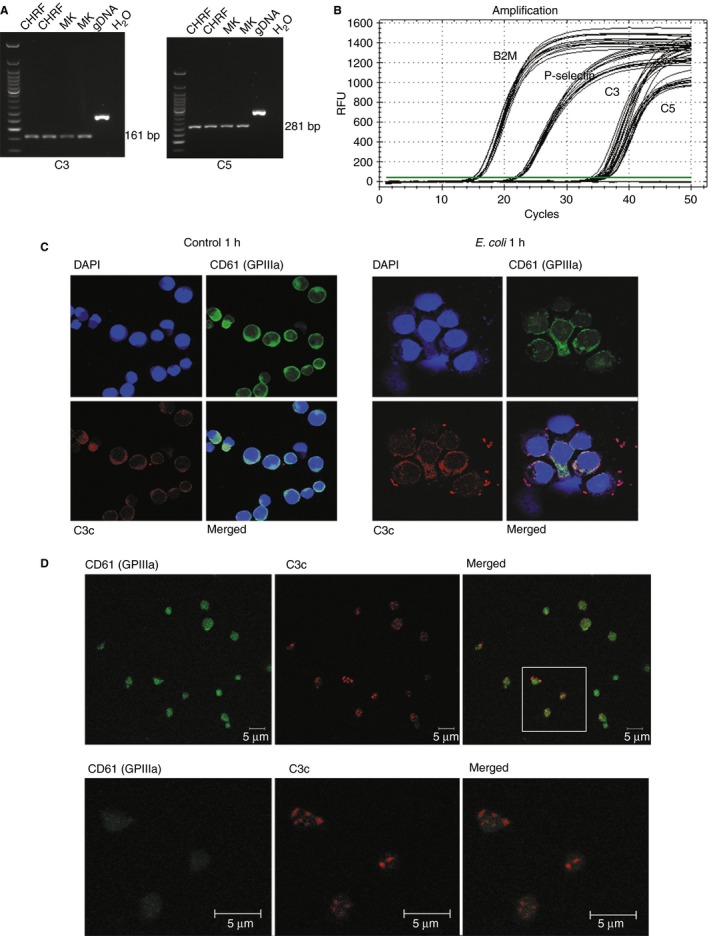
DNA, RNA and protein analysis of C3 and C5 in megakaryocytes and platelets. (A) PCR amplification with intron‐spanning primers shows the presence of complement C3 and C5 mRNA in the megakaryocytic cell line CHFR, as well as in megakaryocytes differentiated in culture. In contrast, unspliced fragments are seen when DNA is used. (B) Real time PCR analysis of C3 and C5 mRNA in apheresis platelets analyzed using Taqman probes indicates the amount of C3 and C5 mRNA. β2‐microglobulin served as reference gene, P‐selectin as positive control. (C) C3 (red) and CD61 (green) are detectable in MKs differentiated in culture. After 1 h incubation with heat inactivated *E. coli* O18:K1 (blue, stained with DAPI), C3 is released and transferred to the bacteria surface. (D) Confocal laser microscopy allows detection of CD61 (green) and C3 (red) in platelet granules of manually isolated platelets. DAPI, 4′,6‐diamidino‐2‐phenylindole; gDNA, genomic DNA; GP, glycoprotein; MK, megakaryocyte; RFU, relative fluorescence units.

PCR analyses with intron‐spanning primers showed the presence of C3 and C5 mRNA in culture‐differentiated megakaryocytes and in the megakaryocytic cell line CHRF (Fig. 3A). Sequence analysis confirmed that the bands corresponded to C3 and C5 (Figs S8 and S9).

Real‐time PCR analyses of RNA isolated from washed platelets showed the presence of C3 and C5 mRNA at low concentrations (Fig. 3B).

### Confocal laser microscopy

Confocal laser microscopy confirmed the presence of complement protein in megakaryocytes and platelets. Complement C3 was observed intracellularly in mature culture‐differentiated megakaryocytes in the absence of other complement sources. The incubation of megakaryocytes with HK *E. coli* O18:K1 led to the release of C3 products that bound to the bacterial surface (Fig. 3C). When unstimulated resting manually isolated platelets were analyzed by confocal microscopy, C3 was identified in granules of platelets (Fig. 3D). Isotype controls are shown in Fig. S10. Incubation of washed platelets with live and HK *E. coli* K12 C600 or *E. coli* O18:K1 did not induce binding of C3 to the bacterial surface (Fig. S11).

### Western blot results for cellular and plasmatic C3 protein

**Figure 4:**
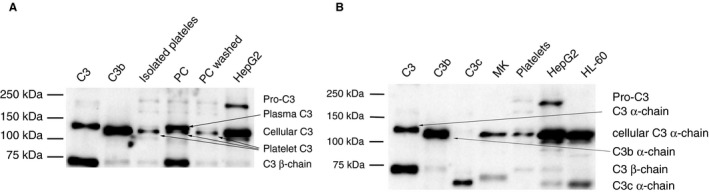
Cellular C3 and plasmatic C3 protein in platelets. Cell lysates were studied by use of an SDS‐PAGE gel with a C3d polyclonal antibody under reducing conditions. (A) Platelets in platelet concentrates (PC) carry C3 from plasma plus platelet C3. Plasma C3 is lost after washing (see PC washed). Lysates of manually isolated platelets, HepG2 cells and purified C3b are shown for comparison. (B) Culture‐differentiated megakaryocytes (MKs), manually isolated platelets, HepG2 cells and HL‐60 cells contain C3 protein. This C3 migrates with a slightly lower molecular mass than purified plasma C3 (first lane). The protein concentrations loaded were: 3.8 μg HepG2 total protein, 4.8 μg HL‐60 total protein, 4 μg of platelet total protein, platelet concentrate (PC) total protein and PC washed total protein, and 8.4 μg of total MK protein. Purified C3b and C3c proteins from plasma are shown for comparison.

We determined the electrophoretic migration behavior of the C3 protein in megakaryocytes, platelets, hepatocytes, and monocytes. With use of an anti‐C3d antibody, the α‐chain of C3 was detectable in all four cell types. The molecular mass was ~ 100 kDa in all cell lysates, and the concentration of C3 was highest in HepG2 cells, followed by HL‐60 cells, platelets, and megakaryocytes. Surprisingly, the molecular mass of plasma C3 was slightly higher than that of cellular C3. The previously reported pro‐C3 with a molecular mass of ~ 180 kDa 39 was only found in hepatocytes. The C3 β‐chain was observed in most samples (Fig. 4).

Western blot analysis of C3 in PCs showed two bands: a larger one, corresponding to plasmatic C3, and a smaller one, migrating with the same size as C3 in all variants of washed platelets as well as in hepatocytes (Fig. 4A). The data suggest that platelets can bind complement C3 from plasma, as previously reported, but they also contain C3 originating from megakaryocytes.

### Bacteria trigger C3 expression in platelets

**Figure 5:**
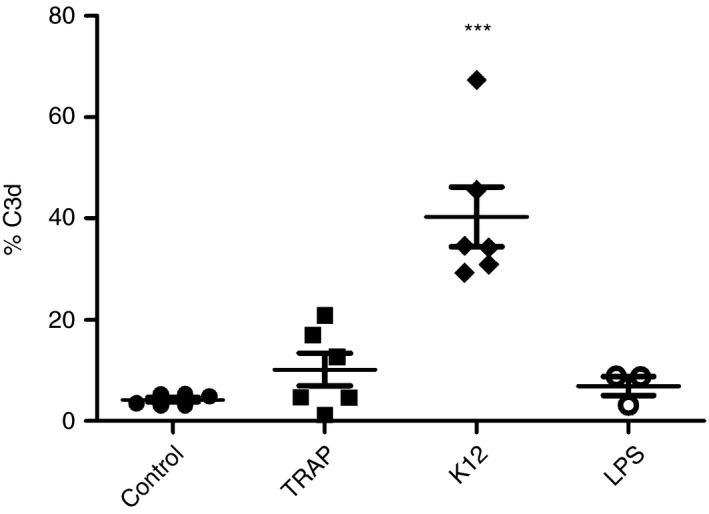
C3 expression in platelets increases with bacterial incubation. Manually isolated platelets were incubated with 14 μm thrombin receptor activating peptide 6 (TRAP‐6), 3 μg mL^−1^ lipopolysaccharide (LPS) from *Escherichia coli* O111:B4 and from live *E. coli* K12 at a ratio of 1 : 10 or SSP+ buffer for 45 min, and stained with C3d antibody. Flow cytometry analysis showed an increase in C3 expression on the surfaces of platelets after incubation with bacteria, but not after incubation with other platelet activators, e.g. TRAP‐6. The level of significance with respect to the controls is ***P < 0.001

We observed a five‐fold increase in C3 expression on the surfaces of manually isolated platelets after exposure of platelets to live *E. coli* K12 C600 for 1 h. TRAP‐6 and 3 μg mL^−1^ LPS from *E. coli* O111:B4 failed to induce comparable upregulation of C3 (Fig. 5). ADP and thrombin also did not upregulate C3 (Fig. S12). A representative scatter plot is shown in Fig. S13.

### Platelets can modify C3 activation caused by *E. coli*

**Figure 6:**
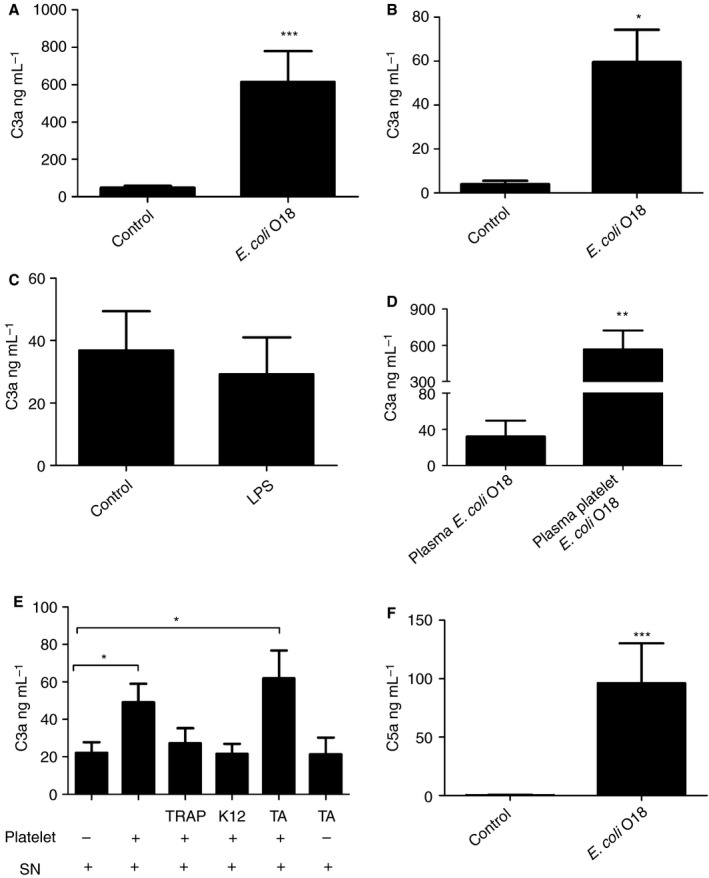
Platelets enhance plasmatic C3 activation caused by *Escherichia coli*. (A, B) Platelet concentrates containing 5% plasma were incubated with heat‐killed (HK) *E. coli* O18:K1 for 3 h, after which C3a was measured by ELISA in (A) releasates (*n* = 12) and (B) lysates (*n* = 4). (C) Lipopolysaccharide (LPS) failed to trigger C3 activation and C3a release. (D) Incubation of plasma with HK *E. coli* O18:K1 for 3 h induced C3a release, which was lower than when plasma containing platelets was incubated (*n* = 7–12, normalized to control). (E) Live *E. coli* K12 was incubated with fresh plasma for 1 h. The supernatant containing activated C3 was recovered, and subsequently incubated for 1 h with resting manually isolated platelets, platelets activated with thrombin receptor activating peptide 6 (TRAP‐6) or *E. coli* K12, and platelets inactivated by theophylline‐adenosine (TA) treatment. Resting and inactivated platelets enhanced the plasmatic activation caused by *E. coli*. (F) Platelet concentrates were incubated with HK *E. coli* O18:K1 for 3 h, and C5a was measured by ELISA. Mean ± standard error of the mean is shown for all graphs. Levels of significance with respect to the controls are as follows: *P < 0.05, *P < 0.01, and ***P < 0.001. PLT, platelet; SN, supernatant.

ELISA analyses of C3a levels showed that incubation of PCs containing ~ 5% plasma with HK *E. coli* O18:K1 resulted in complement activation. C3a activation was high in both platelet releasates (Fig. 6A) and platelet lysates (Fig. 6B). In contrast, incubation of platelets with LPS did not induce C3 activation (Fig. 6C). Whereas incubation of plasma with HK *E. col*i O18:K1 led to only moderate C3 activation (32 ng mL^−1^), the addition of autologous manually isolated platelets increased the C3a release by > 10‐fold (565.6 ng mL^−1^) (Fig. 6D).

Also, the moderate C3 activation by *E. coli* K12 C600 in plasma was measurably enhanced by the addition of platelets. Preactivated platelets (activated by TRAP‐6 or *E. coli* K12 C600) did not lead to an increase in C3a release (Fig. 6E).

Interestingly*, E. coli* O18:K1 also induced C5 activation in PCs, resulting in a 200‐fold increase in released C5a (Fig. 6F). Taken together, these data indicate that platelets promote complement activation in plasma in the presence of *E. coli*.

## Discussion

Most available data regarding interactions of Gram‐negative bacteria with platelets are based on the use of LPS. We studied the interaction of *E. coli* with platelets and the effect on platelet activation. An indicator of platelet activation was the expression of CD62P and CD63. Contact of platelets with *E. coli* leads to rapid platelet activation and release of the contents of α‐granules and dense granules. The results of flow cytometry indicate that *E. coli* can directly interact with and activate platelets in the absence of bridging plasma proteins. Blocking of TLR4 with a specific polyclonal antibody did not inhibit platelet activation by bacteria. *E. coli* O111:B4 LPS failed to trigger platelet activation, which is in accordance with the current literature 13, 16, 40. An RGDS peptide blocking the most abundant platelet GP, α_2b_β_3_ (GPIIbIIIa), also did not affect platelet activation by *E. coli*. Apparently, α_2b_β_3_ is not involved in the interaction of *E. coli* with platelets.

Our results show that heat‐treated complement‐resistant *E. coli* O18:K1 can trigger platelet activation much more effectively than the live bacterial strain. We hypothesize that heat treatment modifies bacterial surface proteins, allowing more efficient interaction between bacteria and platelets.

Pathogenic bacteria have different ways of evading the immune response, such as inhibition of complement activation. Complement‐resistant bacteria with the O antigen restrict alternative complement activation, whereas the K1 antigen inhibits classic complement activation 41, 42. Other bacteria can withstand the complement system effect by binding host complement regulatory molecules such as factor H or C4‐binding protein 43, 44, by preventing bacterial opsonization, or by resisting membrane attack complex (MAC)‐mediated lysis 45.

Previous RNA and protein array studies showed the presence of complement C3 protein and mRNA in platelets 32, 33. However, it was not clarified whether the detected complement had bound to the platelets from plasma and was integrated by pinocytosis. We analyzed the presence of C3 mRNA and protein in megakaryocytes in the absence of any external source of complement C3, and proved its presence. However, our results indicate that, under the chosen conditions, platelets presumably do not synthesize new C3 protein, because no changes in mRNA were observed.

The C3 protein of washed platelets and that of megakaryocytes showed the same mobility on polyacrylamide gels, which differed from that of plasma C3. This strongly suggests that megakaryocytes synthesize C3 and transfer it to platelets during thrombopoiesis. Importantly, unwashed platelets showed two bands: a larger band corresponding to plasmatic complement, and a smaller one corresponding to megakaryocytic C3. Thus, it is highly likely that some platelet C3 comes from megakaryocytes. Whether or not this platelet C3 has any functional importance has not yet been evaluated. We found that C3 stored in the granules of washed platelets can translocate to the platelet surface upon bacteria‐induced platelet activation and degranulation. Translocation of platelet C3 seems to be attributable to an effect of bacteria, as TRAP‐6 and LPS from *E. coli* O111:B4 failed to induce significant upregulation of C3 expression on the platelet surface. There is general agreement that the majority of C3 in plasma originates from hepatocytes 46. Surprisingly, the migration behavior of hepatocytic C3 on polyacrylamide gels corresponds to that of C3 from megakaryocytes, and differs from that of plasmatic C3. Presumably, post‐translational modifications occur during secretion from the liver into the plasma.

Several publications have reported that activated platelets can induce activation of the plasmatic complement system, e.g. via chondroitin sulfate, which is expressed on platelets upon thrombin activation 26. P‐selectin on activated platelets was shown to bind C3b, forming C3 convertase together with factor B, which can trigger formation of the C5b‐9 MAC complex on the platelet surface 25. Properdin on activated platelets can recruit C3(H_2_O)/C3b, forming C3 convertase on the platelet surface in the presence of factor B 47. However, we did not observe induction of platelet complement activation and C3a formation in washed platelets activated by *E. coli*. This lack of activation of platelet‐borne complement is probably attributable to the absence of necessary cofactors or the presence of complement activation inhibitors on the platelet surface. Interestingly, platelets in combination with plasma were able to induce complement activation to a significantly higher extent than plasma alone. Importantly, even though, in plasma, both *E. coli* K12C600 and *E. coli* O18:K1 activated C3 and induced release of C3a, the addition of autologous platelets to plasma increased complement activation.

In conclusion, the contribution of platelets to activation of the complement system could be very important for strengthening innate immune defense against bacteria, as platelets are the first cells to arrive at the site of an injury.

## Addendum

I. Arbesu designed and performed experiments, analyzed the data, and wrote the manuscript. M. Bucsaiova performed experiments. M. B. Fischer provided access to blood and platelet concentrates, and contributed to writing of the manuscript. C. Mannhalter supervised the study, designed experiments, analyzed the data, and contributed to writing of the manuscript.

## Disclosure of Conflict of Interests

The authors state that they have no conflict of interest.

## Supporting information


**Fig. S1.** CD62P and CD63 expression on platelets after incubation with bacteria.
**Fig. S2.** *E. coli* activates platelets independently of TLR4 and GPIIbIIIa.
**Fig. S3.** Scatter graph of platelets incubated withlive *E. coli* K12 and live *E. coli* O18:K1.
**Fig. S4.** Scatter graph of platelets incubated with live *E. coli* K12.
**Fig. S5.** Scatter graph of platelets incubated with live *E. coli* K12 and nTLR4.
**Fig. S6.** Scatter graph of platelets incubated with live *E. col*i K12 and RGDS.
**Fig. S7.** Scatter graph of platelets incubated with HK *E. coli* K12 and HK *E. coli* O18:K1.
**Fig. S8.** Sequencing results of primary megakaryocytes show the presence of C3 mRNA.
**Fig. S9.** Sequencing results of primary megakaryocytes show the presence of C5 mRNA.
**Fig. S10.** Platelets incubated with C3 isotype controls.
**Fig. S11.** Platelets incubated with live and heat‐killed *E. coli* K12 and *E. coli* O18:K1.
**Fig. S12.** C3 expression in platelets incubated with various agonists.
**Fig. S13.** Scatter graph of platelets incubated with live *E. coli*.
**Table S1.** MFI ± SEM and % ± SEM of platelets incubated with TRAP, live *E. coli* K12 and live *E. coli* O18:K1. *n* = 4.
**Table S2.** MFI ± SEM and % ± SEM of platelets incubated with TRAP, live *E. coli* K12 and live *E. coli* O18:K1 in the presence or absence of plasma. *n* = 5.
**Table S3.** MFI ± SEM and % ± SEM of platelets incubated with TRAP, live *E. coli* K12, nTLR4 and AT10. *n* = 4.
**Table S4.** MFI ± SEM and % ± SEM of platelets incubated with TRAP, live *E. coli* K12 and RGDS. *n* = 6.
**Table S5.** MFI ± SEM and % ± SEM of platelets incubated with TRAP, HK *E. coli* K12 and HK *E. coli* O18:K1. *n* = 6.
**Table S6.** Fold change difference of MFI ± SEM and % ± SEM of platelets incubated with TRAP, HK *E. coli* K12 and HK *E. coli* O18:K1, and platelets incubated with TRAP, live *E. coli* K12 and live *E. coli* O18:K1.
**Table S7.** % ± SEM of platelets incubated with TRAP, thrombin, ADP, live *E. coli* K12 and LPS. *n* = 3.
**Table S8.** % ± SEM of platelets incubated with TRAP, live *E. coli* K12 and LPS. *n* = 6.
