# Supplementary material for: Platelet‐borne complement proteins and their role in platelet–bacteria interactions
Source: J Thromb Haemost. 2016 Nov 11;14(11):2241–52. doi: 10.1111/jth.13495 (PMC5299534; doi:10.1111/jth.13495)
Supplement: Supplementary file 1 — Fig. S1. CD62P and CD63 expression on platelets after incubation with bacteria. Fig. S2. E. coli activates platelets independently of TLR4 and GPIIbIIIa. Fig. S3. Scatter graph of platelets incubated withlive E. coli K12 and live E. coli O18:K1. Fig. S4. Scatter graph of platelets incubated with live E. coli K12. Fig. S5. Scatter graph of platelets incubated with live E. coli K12 and nTLR4. Fig. S6. Scatter graph of platelets incubated with live E. coli K12 and RGDS. Fig. S7. Scatter graph of platelets incubated with HK E. coli K12 and HK E. coli O18:K1. Fig. S8. Sequencing results of primary megakaryocytes show the presence of C3 mRNA. Fig. S9. Sequencing results of primary megakaryocytes show the presence of C5 mRNA. Fig. S10. Platelets incubated with C3 isotype controls. Fig. S11. Platelets incubated with live and heat‐killed E. coli K12 and E. coli O18:K1. Fig. S12. C3 expression in platelets incubated with various agonists. Fig. S13. Scatter graph of platelets incubated with live E. coli. Table S1. MFI ± SEM and % ± SEM of platelets incubated with TRAP, live E. coli K12 and live E. coli O18:K1. n = 4. Table S2. MFI ± SEM and % ± SEM of platelets incubated with TRAP, live E. coli K12 and live E. coli O18:K1 in the presence or absence of plasma. n = 5. Table S3. MFI ± SEM and % ± SEM of platelets incubated with TRAP, live E. coli K12, nTLR4 and AT10. n = 4. Table S4. MFI ± SEM and % ± SEM of platelets incubated with TRAP, live E. coli K12 and RGDS. n = 6. Table S5. MFI ± SEM and % ± SEM of platelets incubated with TRAP, HK E. coli K12 and HK E. coli O18:K1. n = 6. Table S6. Fold change difference of MFI ± SEM and % ± SEM of platelets incubated with TRAP, HK E. coli K12 and HK E. coli O18:K1, and platelets incubated with TRAP, live E. coli K12 and live E. coli O18:K1. Table S7. % ± SEM of platelets incubated with TRAP, thrombin, ADP, live E. coli K12 and LPS. n = 3. Table S8. % ± SEM of platelets incubated with TRAP, live E. coli K12 and LPS. n = 6. [file JTH-14-2241-s001.pdf]

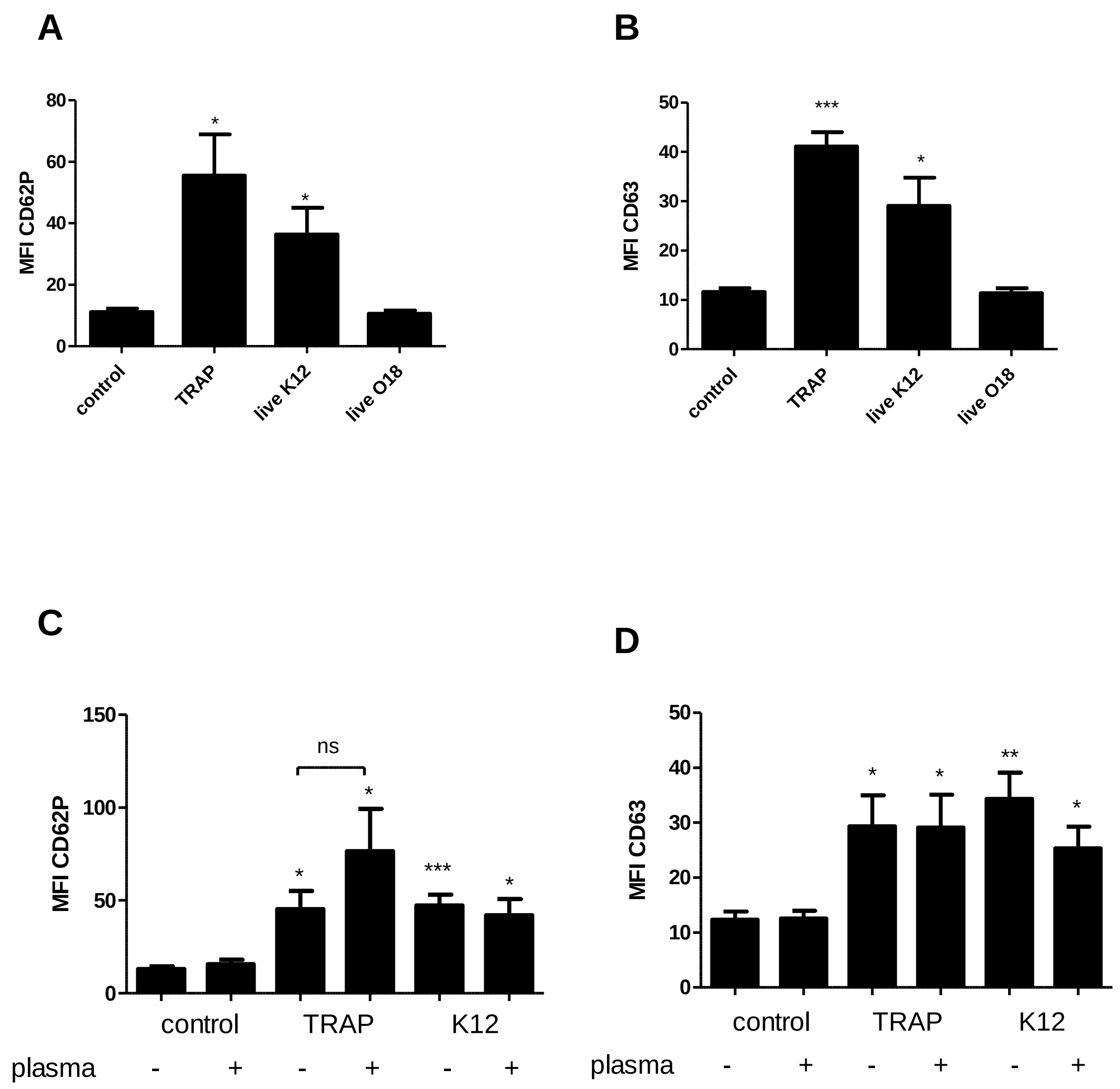

Suppl Figure 1. CD62P and CD63 expression on platelets after incubation with bacteria. CD62P (A) and CD63 (B) were detected in PRP platelets by flow cytometry, after gating for CD41+ platelets which were unstimulated, stimulated with TRAP, live *E. coli K12* or live *E. coli O18:K1* for 45 minutes (n=4). CD62P (C) and CD63 (D) expression in unstimulated manually isolated platelets, stimulated with TRAP, live *E. coli K12* or live *E. coli O18:K1* for 45 minutes were also analyzed in the presence or absence of 5% autologous plasma (n=5). The data represents MFI (mean ± SEM) of independent experiments. Levels of significance with respect to the controls are marked as *p <0.05, *p <0.01 and ***p<0.001.

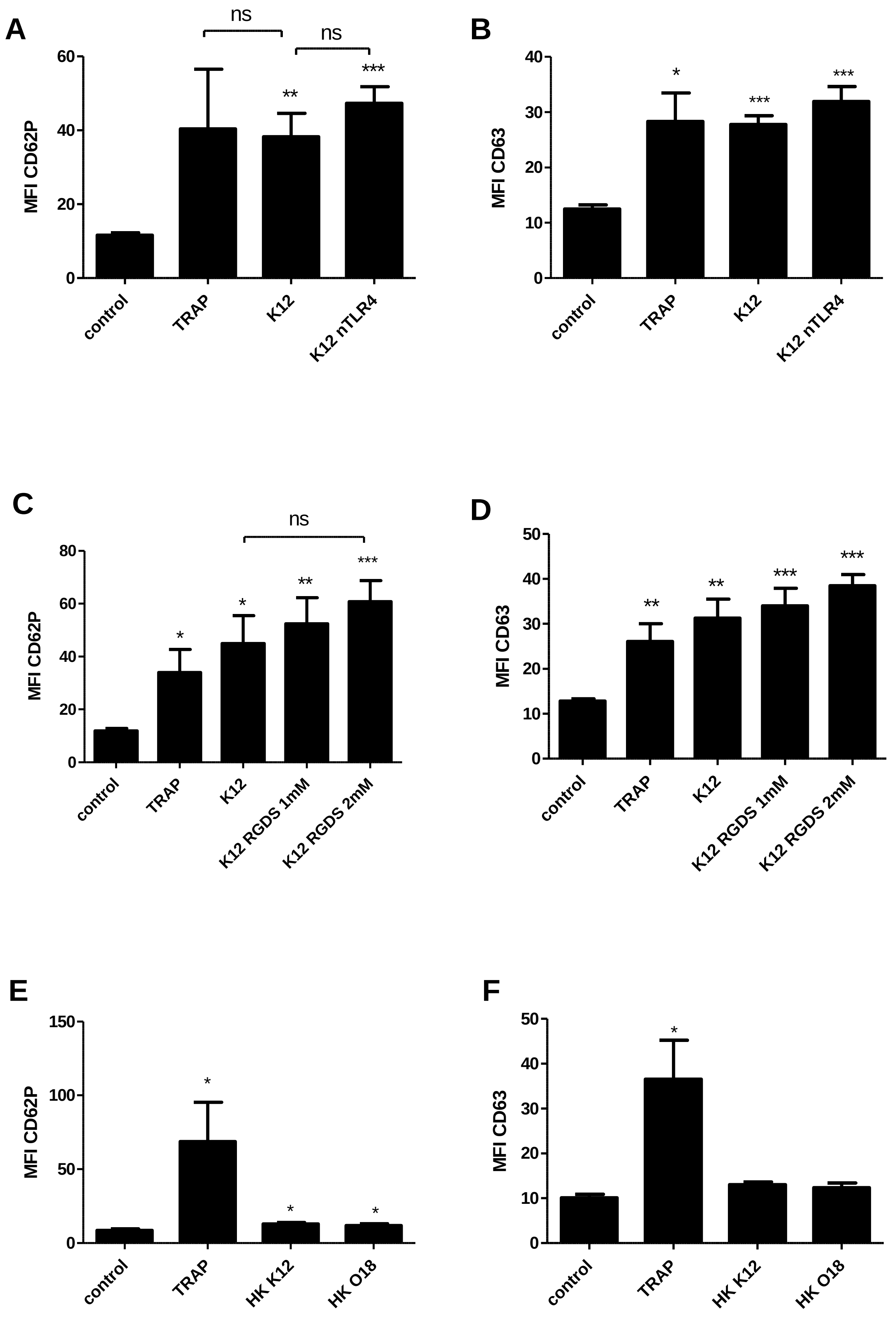

Suppl Figure 2. *E. coli* activate platelets independently of TLR4 and GPIIbIIIa.

Platelets were incubated with 20 µg/ml neutralizing polyclonal TLR4 antibody for 20 minutes prior to the stimulation with live *E. coli K12*. Following stimulation with buffer, TRAP or live *E. coli K12*, CD62P (A) and CD63 (B) expression was analyzed by flow cytometry (n=4). Data shows % (mean ± SEM).

Platelets were also incubated with 1-2 mM RGDS peptide to block the GPIIbIIIa receptor before the stimulation with live *E.coli K12*. Platelets were not stimulated, stimulated with TRAP or live *E. coli K12*. CD62P (C) and CD63 (D) expression was subsequently analyzed (n=6). Bars represent MFI (mean ± SEM).

PRP platelets were incubated with HK *E. coli K12* and HK *E.coli O18:K1* and compared to unstimulated and TRAP stimulated platelets. CD62P (E) and CD63 (F) results are shown. Data represents % (mean ± SEM) of 6 independent experiments. Levels of significance with respect to the controls are marked as *p <0.05, *p <0.01 and ***p<0.001.

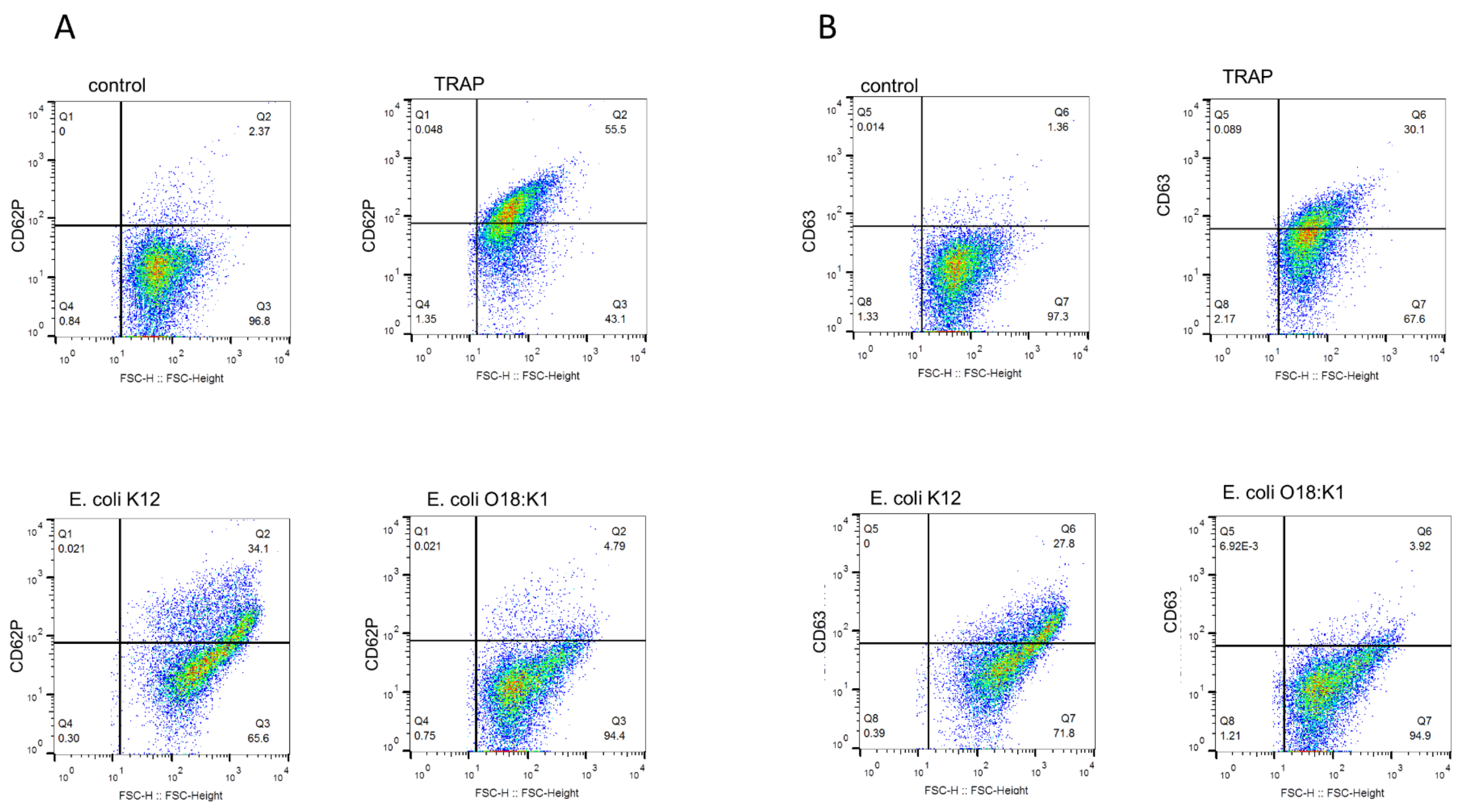

Suppl Figure 3. Scatter graph of platelets incubated with live *E. coli K12* and live *E. coli O18:K1*.

Representative scatter graph of PRP platelets incubated with TRAP, live *E. coli K12* and live *E. coli O18:K1*, showing CD62P-PE (A) and CD63-FITC (B) after gating for CD41+.

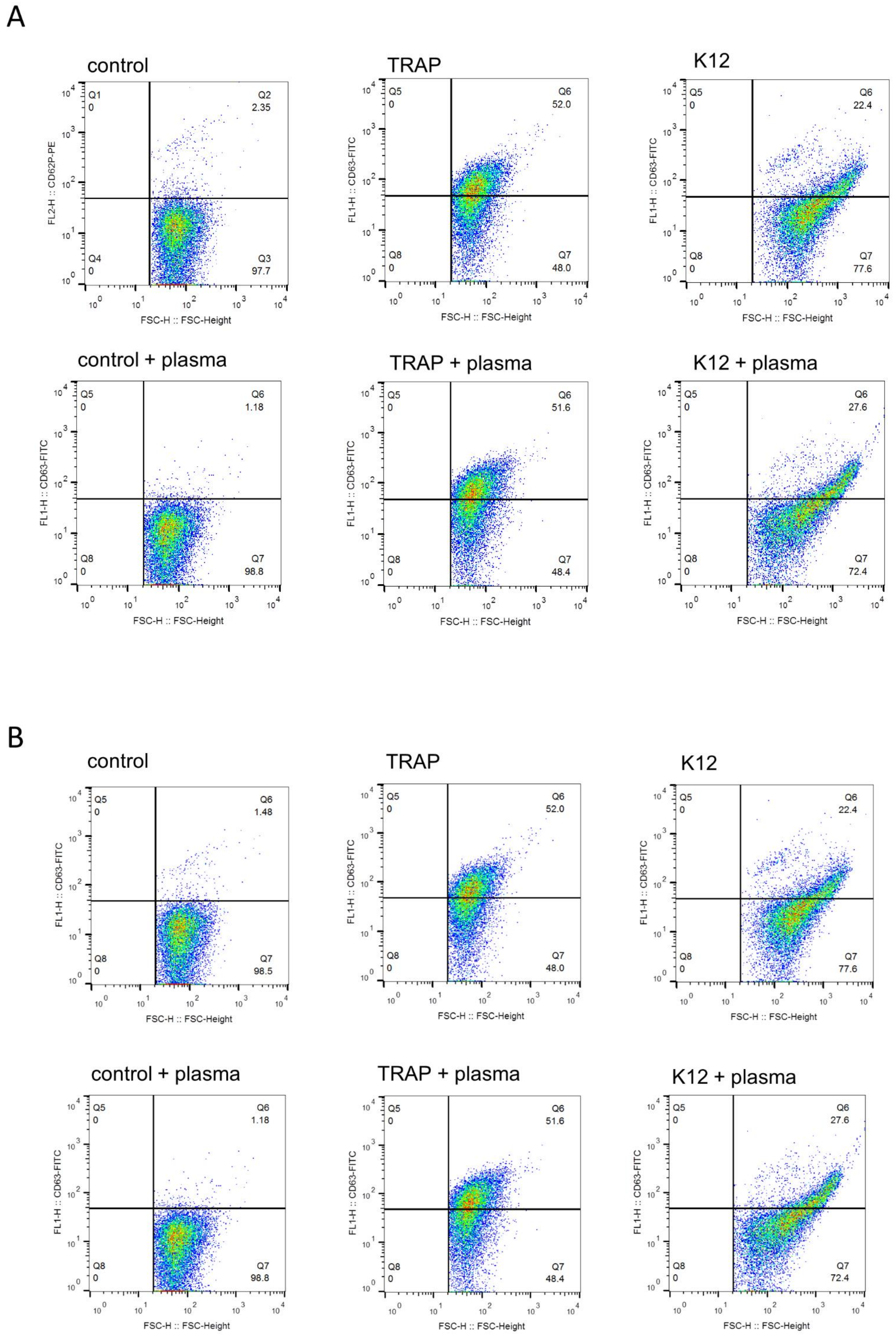

Suppl Figure 4. Scatter graph of platelets incubated with live *E. coli K12*.

Representative scatter graph of washed isolated platelets incubated with TRAP, live *E. coli K12* in the presence and absence of 5% autologous plasma, showing CD62P-PE (A) and CD63-FITC (B) after gating for CD41+.

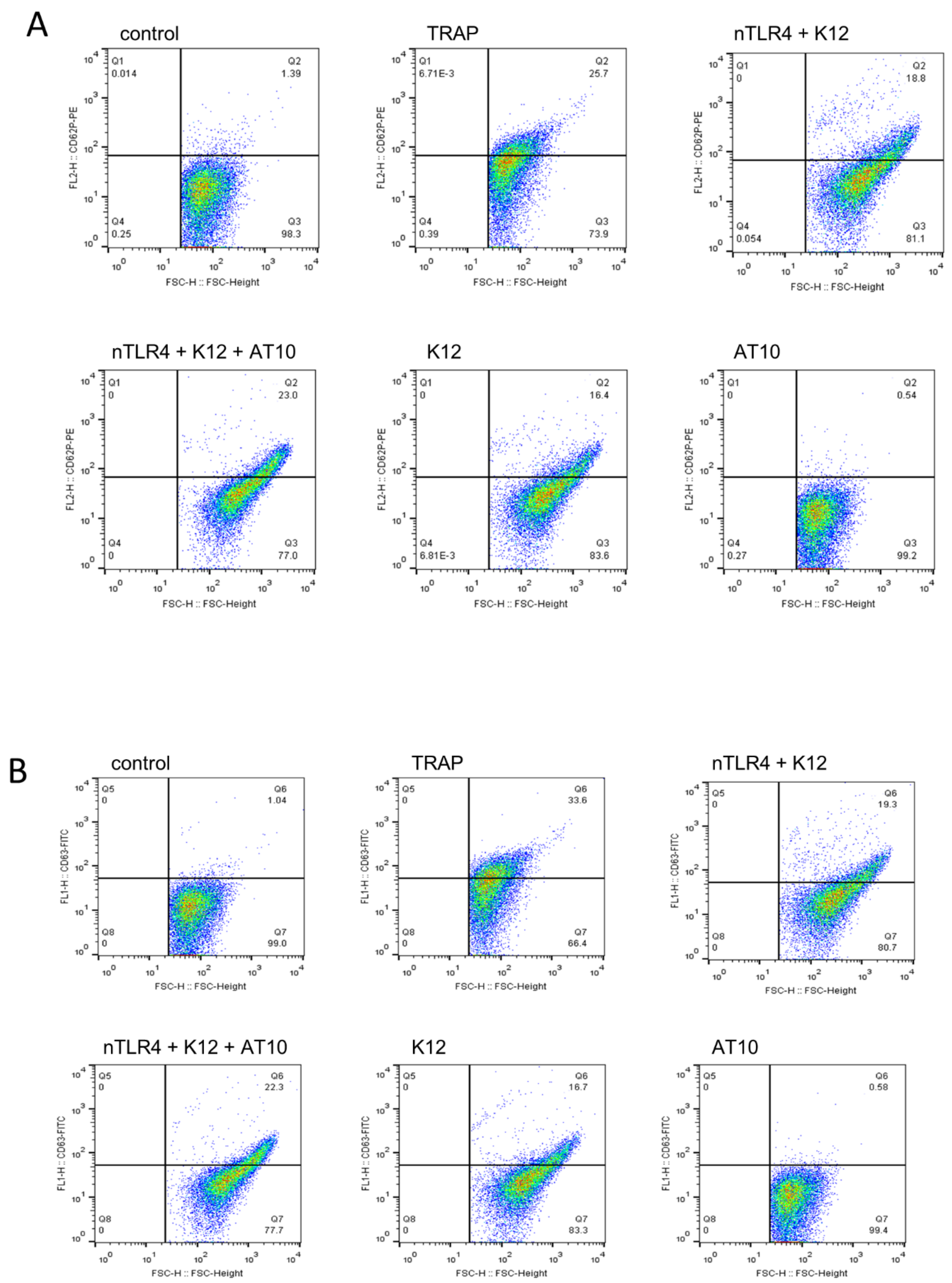

Suppl Figure 5. Scatter graph of platelets incubated with live *E. coli K12* and nTLR4.

Representative scatter graph of platelets incubated with TRAP, live *E. coli K12*, blocking nTLR4 antibody, and AT10, showing CD62P-PE (A) and CD63-FITC (B) after gating for CD41+.

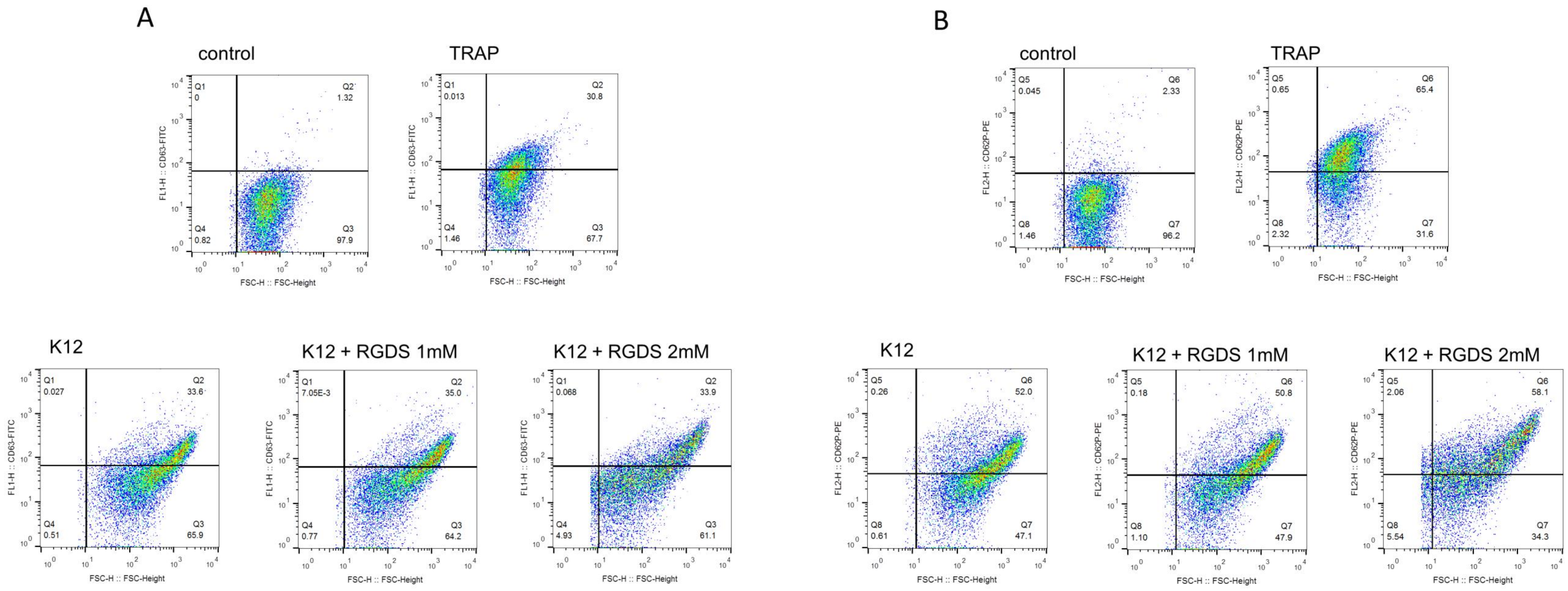

Suppl Figure 6. Scatter graph of platelets incubated with live *E. coli K12* and RGDS.

Representative scatter graph of platelets incubated with TRAP, live *E. coli K12* and RGDS blocking peptide, showing CD62P-PE (A) and CD63-FITC (B) after gating for CD41+.

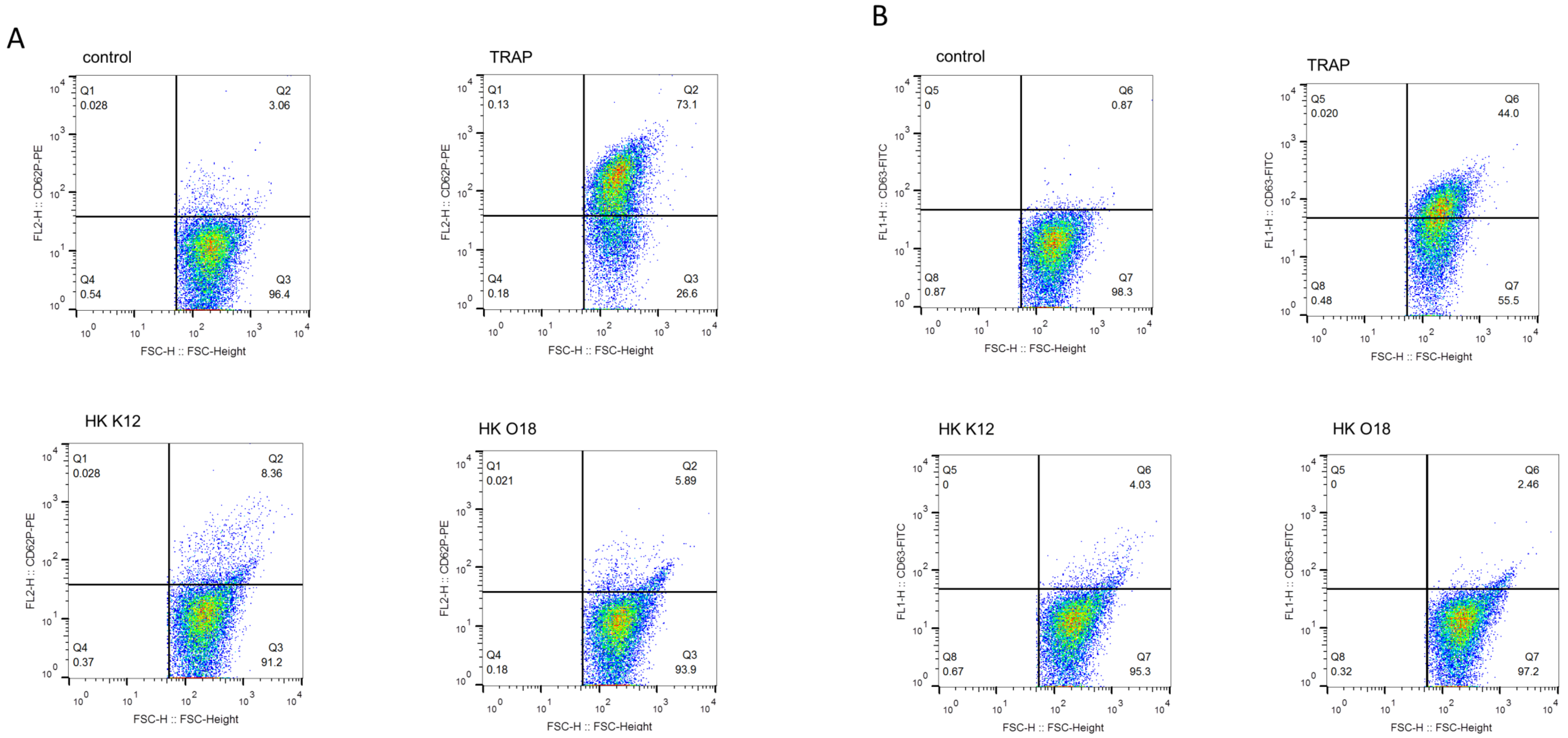

Suppl Figure 7. Scatter graph of platelets incubated with HK *E. coli K12* and HK *E. coli O18:K1*.

Representative scatter graph of platelets incubated with TRAP, HK *E. coli K12* and HK *E. coli O18:K1*, showing CD62P-PE (A) and CD63-FITC (B) after gating for CD41+.

Suppl Table 1. MFI ± SEM and % ± SEM of platelets incubated with TRAP, live E. coli K12 and live E. coli O18:K1. n=4

	MFI CD62P ± SEM	p value	MFI CD63 ± SEM	p value	% CD62P ± SEM	p value	% CD63 ± SEM	p value
control	11.22 ± 1.097	-	11.65 ± 0.7643	-	3.563 ± 0.3487	-	2.630 ± 0.4216	-
TRAP	55.58 ± 13.34	0.0161	41.16 ± 2.878	< 0.0001	48.14 ± 11.53	0.0083	36.34 ± 6.086	0.0015
K12	36.39 ± 8.622	0.0275	29.10 ± 5.670	0.0225	38.54 ± 12.40	0.0304	28.38 ± 8.282	0.021
O18	10.67 ± 1.028	0.727	11.41 ± 0.9788	0.8501	6.673 ± 1.812	0.143	4.970 ± 0.876	0.0528

Suppl Table 2. MFI ± SEM and % ± SEM of platelets incubated with TRAP, live *E. coli K12* and live *E. coli O18:K1* in presence or absence of plasma. n=5

	MFI CD62P ± SEM	p value	MFI CD63 ± SEM	p value	% CD62P ± SEM	p value	% CD63 ± SEM	p value
control	13.00 ± 1.539	-	12.37 ± 1.497	-	3.270 ± 0.244	-	2.042 ± 0.365	-
control plasma	15.80 ± 2.426	0.358	12.62 ± 1.356	0.903	2.786 ± 0.356	0.294	1.668 ± 0.194	0.392
TRAP	45.48 ± 9.595	-	29.36 ± 5.610	-	32.84 ± 9.629	-	23.18 ± 8.384	-
TRAP plasma	76.58 ± 22.77	0.244	29.14 ± 5.978	0.979	43.80 ± 13.38	0.525	25.55 ± 8.189	0.845
K12	47.30 ± 5.830	-	34.34 ± 4.775	-	34.92 ± 4.701	-	24.64 ± 1.155	-
K12 plasma	42.02 ± 8.789	0.630	25.34 ± 3.911	0.183	29.83 ± 7.745	0.590	25.63 ± 6.158	0.879

Suppl Table 3. MFI ± SEM and % ± SEM of platelets incubated with TRAP, live *E. coli K12*, nTLR4 and AT10. n=4

	MFI CD62P ± SEM	p value	MFI CD63 ± SEM	p value	% CD62P ± SEM	p value	% CD63 ± SEM	p value
control	12.20 ± 0.4726	-	10.65 ± 0.9067	-	3.220 ± 0.6592	-	3.073 ± 0.6682	-
TRAP	54.03 ± 9.145	0.0103	43.17 ± 4.638	0.0023	42.63 ± 4.388	0.0009	50.83 ± 2.850	< 0.0001
nTLR4 + K12	35.10 ± 1.790	0.3822*	26.17 ± 2.307	0.6844*	28.07 ± 2.565	0.2502*	29.40 ± 1.756	0.2084*
AT10 + nTLR4 + K12	30.33 ± 0.6119	0.3812*	23.50 ± 0.5033	0.6074*	21.73 ± 2.839	0.3969*	24.37 ± 3.014	0.4781*
K12	32.43 ± 2.046	0.0006	24.77 ± 2.219	0.0042	24.50 ± 0.6807	< 0.0001	26.73 ± 0.2906	< 0.0001
AT10	11.03 ± 0.3528	0.119	9.487 ± 0.7226	0.3712	1.277 ± 0.3973	0.065	1.547 ± 0.2444	0.0985

* p value when compared to K12

Suppl Table 4. MFI ± SEM and % ± SEM of platelets incubated with TRAP, live *E. coli K12* and RGDS. n=6

	MFI CD62P ± SEM	p value	MFI CD63 ± SEM	p value	% CD62P ± SEM	p value	% CD63 ± SEM	p value
control	11.90 ± 0.933	-	12.84 ± 0.491	-	3.640 ± 1.333	-	2.885 ± 0.595	-
TRAP	34.07 ± 8.605	0.028	26.09 ± 3.944	0.0076	25.97 ± 9.437	0.0412	21.24 ± 5.148	0.0053
K12	44.97 ± 10.51	0.0106	31.25 ± 4.229	0.0015	34.97 ± 6.582	0.0009	27.16 ± 4.251	0.0002
RGDS 1mM	52.43 ± 9.78	0.0021	34.00 ± 3.931	0.0003	40.24 ± 5.339	< 0.0001	30.33 ± 4.080 6	< 0.0001
RGDS 2 mM	60.81 ± 7.87	0.0001	38.47 ± 2.511	< 0.0001	47.23 ± 6.900	0.0001	34.02 ± 2.968	< 0.0001

Suppl Table 5. MFI ± SEM and % ± SEM of platelets incubated with TRAP, HK *E. coli K12* and HK *E.coli O18:K1*. n=6

	MFI CD62P ± SEM	p value	MFI CD63 ± SEM	p value	% CD62P ± SEM	p value	% CD63 ± SEM	p value
control	8.585 ± 1.007	-	10.09 ± 0.8201	-	3.617 ± 0.6829	-	1.736 ± 0.2522	-
TRAP	68.63 ± 26.74	0.0486	36.57 ± 8.683	0.0126	53.93 ± 11.13	0.0011	36.87 ± 7.554	0.0023
HK K12	12.91 ± 1.055	0.0142	13.08 ± 0.5522	0.0128	11.99 ± 2.352	0.0066	5.687 ± 0.8235	0.0023
HK O18	11.96 ± 1.050	0.0429	12.37 ± 1.038	0.1158	8.345 ± 1.841	0.0368	3.816 ± 0.6778	0.0262

Suppl Table 6. Fold change difference of MFI ± SEM and % ± SEM of platelets incubated with TRAP, HK *E. coli K12* and *HK E.coli O18:K1*, and platelets incubated with TRAP, live *E. coli K12* and live *E. coli O18:K1*.

	fold MFI CD62P	fold MFI CD63	fold % CD62P	fold % CD63
**HK K12**	1.50	1.30	3.31	3.28
**HK O18**	1.39	1.23	2.31	2.20
**live K12**	3.24	2.50	10.82	10.79
**live O18**	0.95	0.98	1.87	2.02

data are fold change of MFI and % *vs* their respective controls

Suppl Table 7. % ± SEM of platelets incubated with TRAP, thrombin, ADP, live *E.coli K12* and LPS. n=3

	% C3d ± SEM	p value
control	4.205 ± 0.4407	-
TRAP	10.19 ± 3.209	0.0944
K12	40.32 ± 5.877	0.0001
LPS	6.900 ± 1.876	0.0931

Suppl Table 8. % ± SEM of platelets incubated with TRAP, live *E.coli K12* and LPS. n=6

	% C3d ± SEM	p value
control	4.205 ± 0.4407	-
TRAP	10.19 ± 3.209	0.0944
K12	40.32 ± 5.877	0.0001
LPS	6.900 ± 1.876	0.0931

Homo sapiens complement component 3 (C3), mRNA
Sequence ID: ref|NM_000064.3|Length: 5148Number of Matches: 1
Related Information
Range 1: 1135 to 1261GenBankGraphics Next Match Previous Match

Alignment statistics for match #1

Score	Expect	Identities	Gaps	Strand
222 bits(120)	9e-55	125/127(98%)	2/127(1%)	Plus/Plus

```
Query  9     ATCGTGA-CTCT-CCTACCAGATCCACTTCACCAAGACACCCAAGTACTTCAAACCAGGA  66
             ||||||| |||| ||||||||||||||||||||||||||||||||||||||||||||||||
Sbjct  1135  ATCGTGACCTCTCCCTACCAGATCCACTTCACCAAGACACCCAAGTACTTCAAACCAGGA  1194

Query  67    ATGCCCTTTGACCTCATGGTGTTCGTGACGAACCCTGATGGCTCTCCAGCCTACCGAGTC  126
             ||||||||||||||||||||||||||||||||||||||||||||||||||||||||||||
Sbjct  1195  ATGCCCTTTGACCTCATGGTGTTCGTGACGAACCCTGATGGCTCTCCAGCCTACCGAGTC  1254

Query  127   CCCGTGG  133
             |||||||
Sbjct  1255  CCCGTGG  1261
```

Figure 8. Sequencing results of primary megakaryocytes positively show the presence of C3 mRNA.

Homo sapiens complement component 5 (C5), RefSeqGene (LRG_28) on chromosome 9
Sequence ID: ref|NG_007364.1|Length: 104942Number of Matches: 2
Related Information
Range 1: 37447 to 37636GenBankGraphics Next Match Previous Match

Alignment statistics for match #1

Score	Expect	Identities	Gaps	Strand
335 bits(181)	1e-90	187/190(98%)	0/190(0%)	Plus/Minus

```
Query  66     ACCTGGAGCTGGTTGCCACATTTTTCTTCAATATTTAACCAGACTGAATCAAACACTAAT  125
              ||||||||||||||||||||||||||||||||||||||||||||||||||| ||||||||
Sbjct  37636  ACCTGGAGCTGGTTGCCACATTTTTCTTCAATATTTAACCAGACTGAATCAGACACTAAT  37577

Query  126    TCTGCTGTCTGTTCTCCTGTGACGATATAATAGACCAAAAGTCGGGATGAAGGAACCATG  185
              |||||||||||||||||||||||||| |||||||||| ||||||||||||||||||||||
Sbjct  37576  TCTGCTGTCTGTTCTCCTGTGACGATGTAATAGACCAGAAGTCGGGATGAAGGAACCATG  37517

Query  186    TTCTGTGTTACTGGAATGTTTATACTTTGATAAGATGCATCTGAAAATTTCTCCCTCGTG  245
              ||||||||||||||||||||||||||||||||||||||||||||||||||||||||||||
Sbjct  37516  TTCTGTGTTACTGGAATGTTTATACTTTGATAAGATGCATCTGAAAATTTCTCCCTCGTG  37457

Query  246    CCAAAGTGGA  255
              ||||||||||
Sbjct  37456  CCAAAGTGGA  37447
```

Figure 9. Sequencing results of primary megakaryocytes show the presence of C5 mRNA.

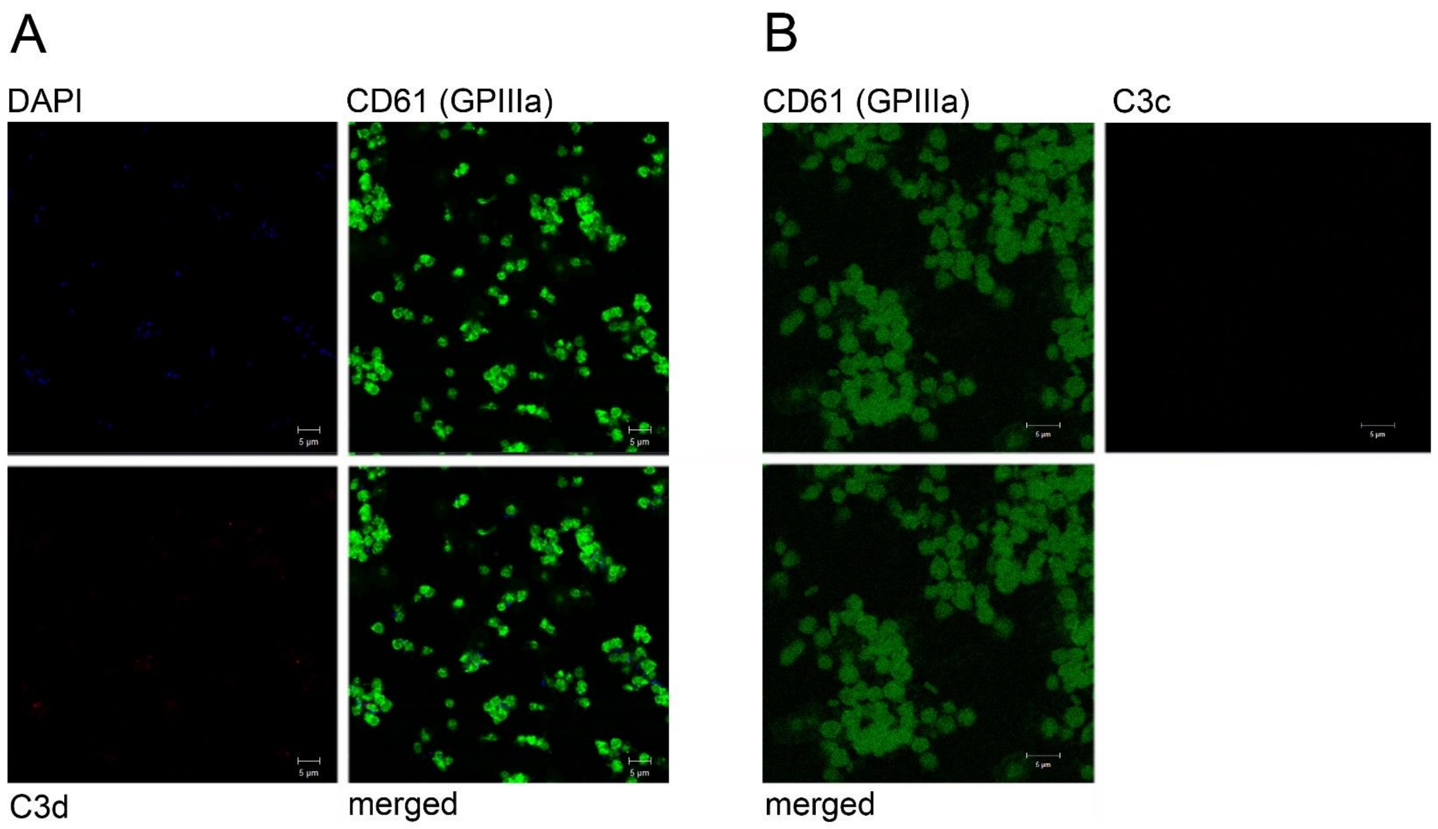

Suppl Figure 10. Platelets incubated with C3 isotype controls.

Platelets incubated with CD61 antibody (green) and the respective C3 isotype controls show the absence of detectable C3d (A) or C3c signals (B).

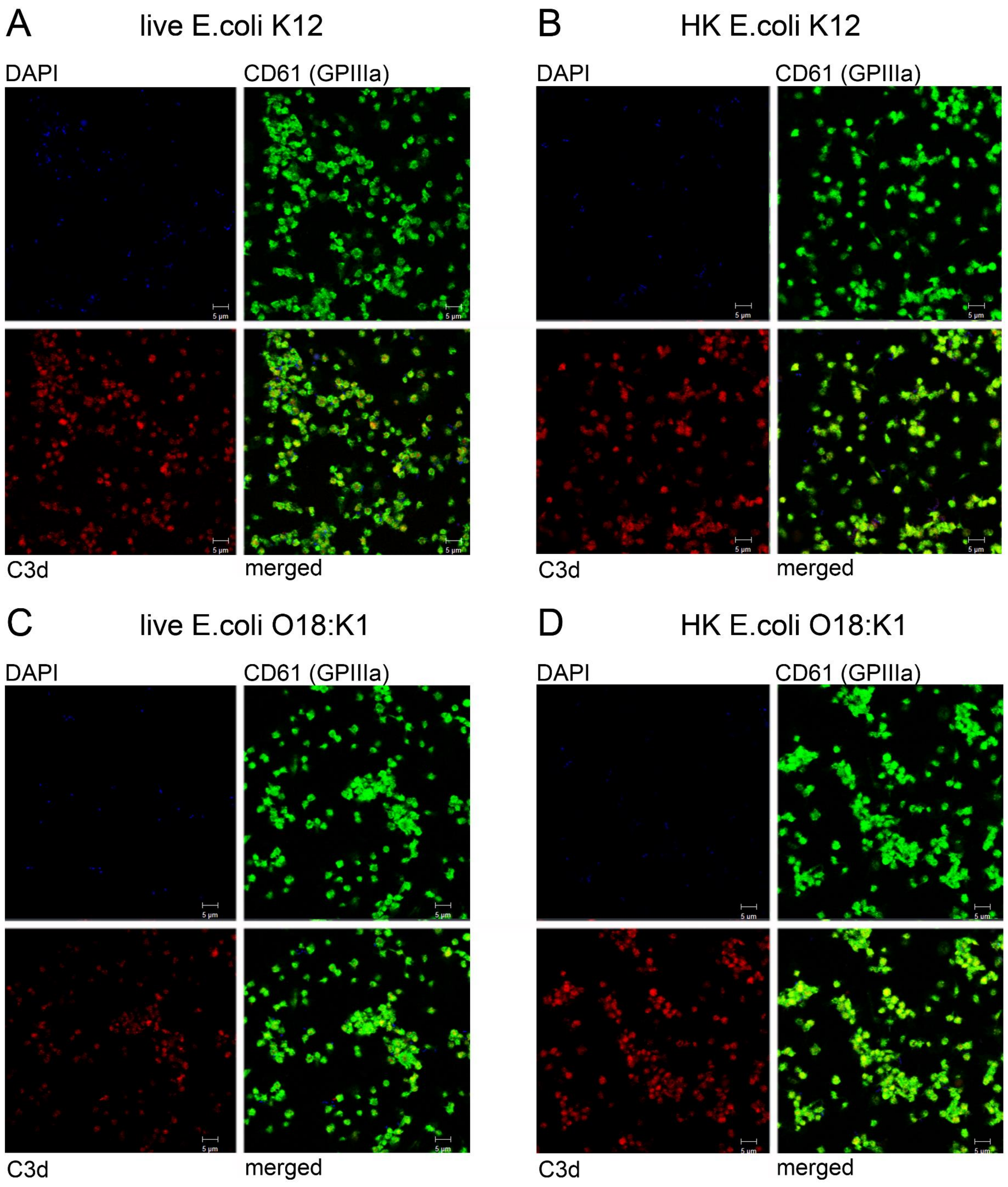

Suppl Figure 11. Platelets incubated with live and heat killed *E. coli K12* and *E. coli O18:K1*

Manually isolated platelets were incubated with live and heat killed *E. coli K12* (A-B) and *E. coli O18:K1* (C-D) and stained with DAPI (blue), CD61 (green) and C3d (red). After 3 hours, no C3 was observed binding to the bacteria.

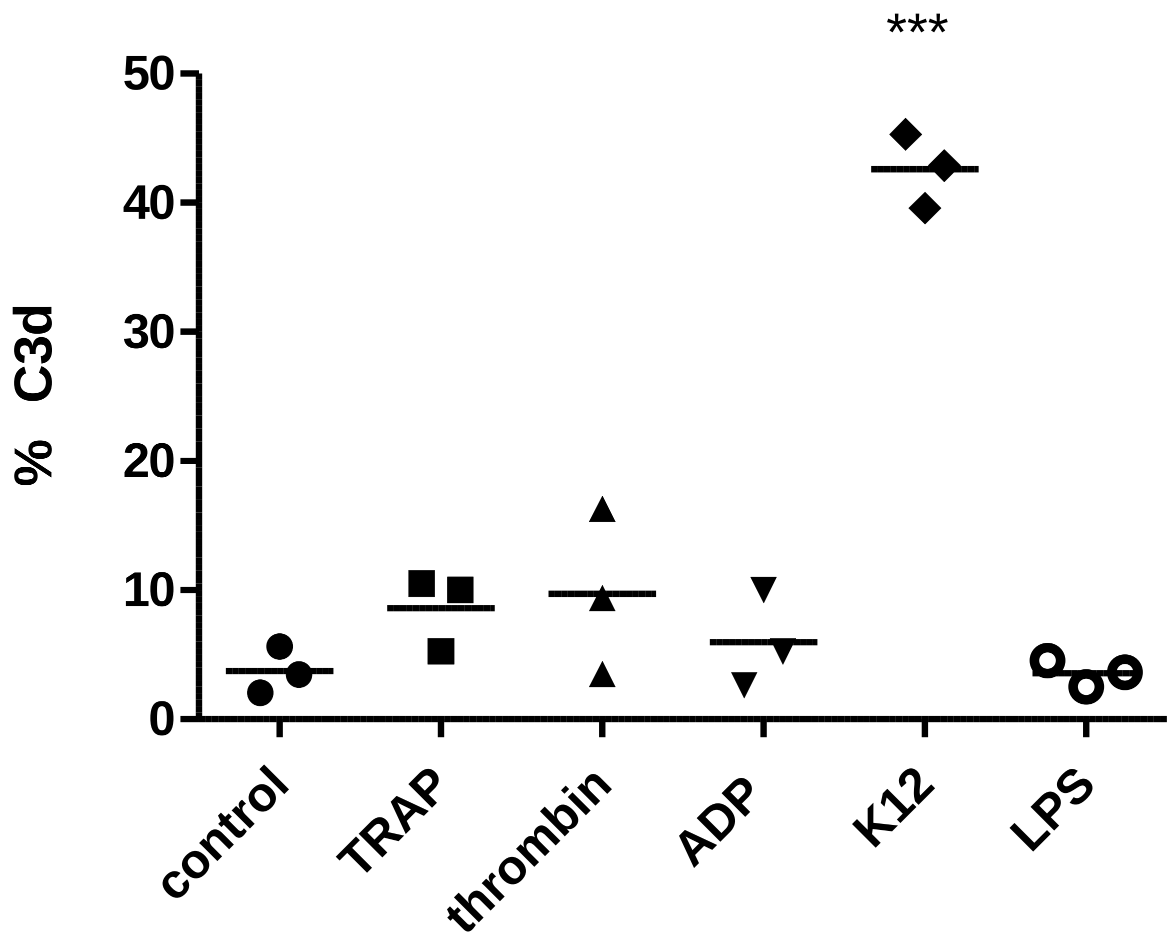

Suppl Figure 12. C3 expression in platelets incubated with various agonists.

Platelets were incubated with TRAP, thrombin, ADP, LPS or *E. coli K12* for 45 minutes and stained with C3d antibody. Flow cytometry analysis shows an increase of C3 on the surface of the platelets after incubation with bacteria, but not after incubation with other platelet activators e.g. TRAP, thrombin. Levels of significance with respect to the controls are marked as ***p<0.001.

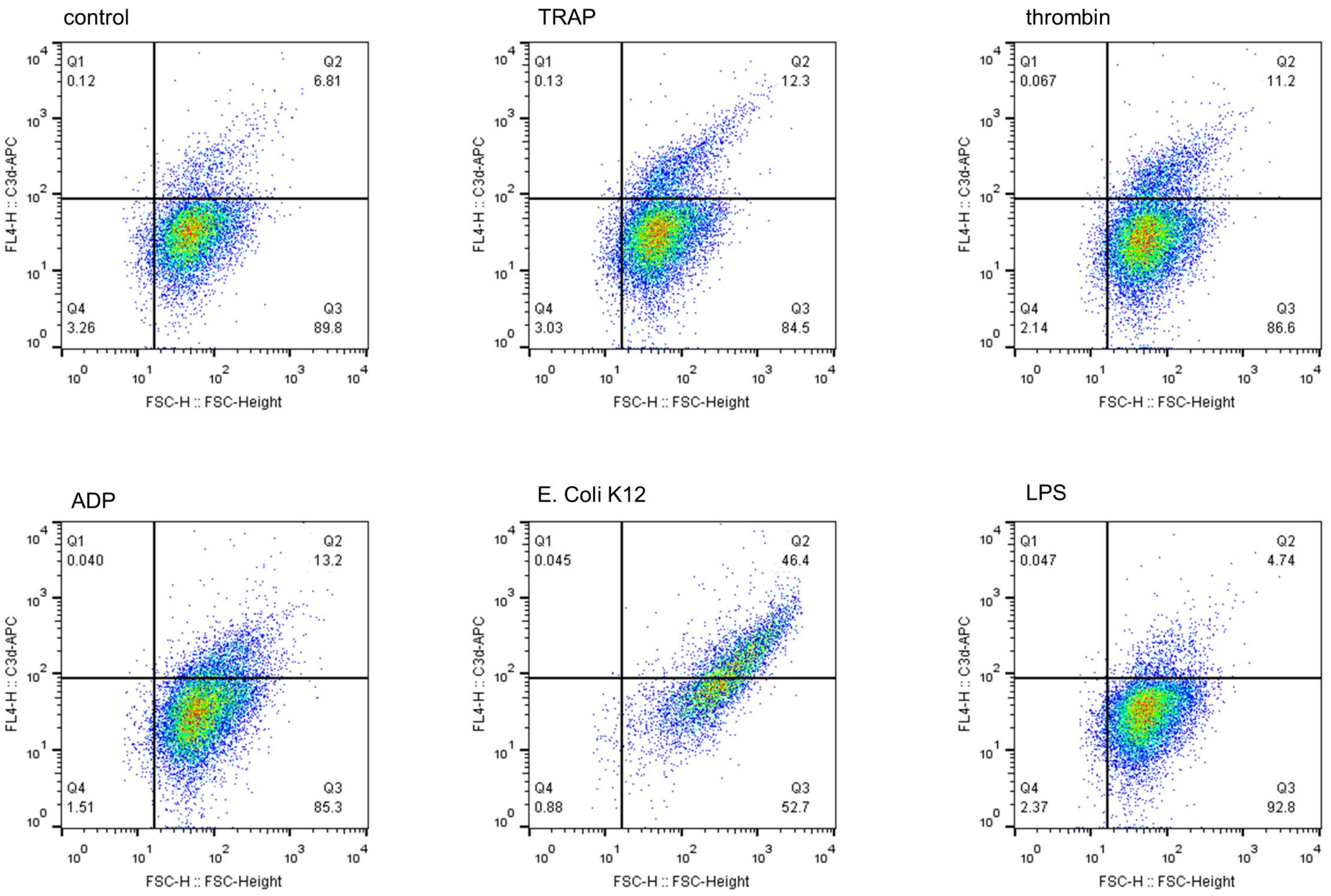

Suppl Figure 13. Scatter graph of platelets incubated with live *E. coli.*

Representative scatter graph of platelets incubated with TRAP, thrombin, ADP, live *E. coli K12* and LPS, showing C3d-APC after gating for CD41+.
